# Height, weight, and body mass index in patients with familial dysautonomia

**DOI:** 10.1371/journal.pone.0293800

**Published:** 2023-11-09

**Authors:** Maria L. Cotrina, Barr Morgenstein, Miguel Perez, Lucy Norcliffe-Kaufmann, Jose-Alberto Palma, Horacio Kaufmann

**Affiliations:** Department of Neurology, Dysautonomia Center, New York University Grossman School of Medicine, New York, New York, United States of America; Human Genetics and Genome Research Institute, National Research Centre, EGYPT

## Abstract

**Background:**

Children with familial dysautonomia (FD) are smaller and grow more slowly than the general population. It is unknown whether this abnormal growth is due to comorbidities that patients with FD live with, or if it is a direct effect of the disease-causing homozygous *ELP-1* mutations. Here, we created growth curves for weight, height, and body mass index (BMI) in male and female children with FD to monitor the nutritional status of patients with FD.

**Methods:**

We used the New York University (NYU) FD Registry which includes data from 680 individuals with FD who were followed longitudinally since birth. We generated sex-specific FD growth charts for three age ranges (birth to 36 months, 2 to 20 years, and 2 to 40 years) and compared them to the general population. We generated Kaplan-Meier curves to test the hypothesis that FD patients with low BMI had shorter survival than the rest of the cohort.

**Results:**

Growth charts generated from 591 individuals with FD show that these patients grow more slowly, reach less height, and gain less weight than the general population. The impact of FD on height was more pronounced in girls than in boys. However, both groups showed markedly low weights, which resulted in low BMI. Low weight, but not height, is already evident at birth. In a subpopulation of FD patients, we found that treatment with growth hormone or spinal fusion surgery helped patients achieve the expected growth characteristic of FD patients, but these treatments did not lead FD patients to achieve the growth pattern of the general population. Contrary to our hypothesis, low BMI had no impact on patient survival.

**Conclusions:**

Pediatric patients with FD have lower height, weight, and BMI compared to the general pediatric population, but this does not appear to affect survival. Growth curves specific to the FD population are an important tool to monitor growth and nutritional status in pediatric patients with FD when the general population growth curves are of limited use.

## Introduction

Familial dysautonomia (FD, OMIM #223900), also known as Riley–Day syndrome, is a rare hereditary sensory and autonomic neuropathy (HSAN-3) first described in 1949 in children of Jewish-Ashkenazi ancestry [1]. It is caused by a founder mutation in the elongator complex protein 1 gene (*ELP1*, OMIM #603722, also known as *IKBKAP*), resulting in impaired development of sensory and afferent autonomic nerves [2]. This mutation originated in Eastern Europe and has a carrier rate as high as 1 in 18 individuals of Ashkenazi ancestry [3].

Patients with FD have a complex phenotype. They have reduced pain and temperature sensation, and suffer frequent “hyperadrenergic autonomic crises” with retching, vomiting, hypertension, and tachycardia that can last from minutes to days. Patients have afferent baroreflex failure that causes neurogenic orthostatic hypotension and extreme blood pressure variability that contributes to chronic kidney disease. All patients experience progressive sensory ataxia and loss of vision [3]. Morbidity and mortality in this population are frequently associated with sleep-disordered breathing, chronic lung disease, and sudden unexpected death during sleep (SUDS) [4].

Patients with FD also have frequent gastrointestinal complaints, which have a significant impact on the quality of life [5]. Neurogenic dysphagia and gastroesophageal reflux are among the first symptoms of the disease [6–8], and result in significant morbidity due to recurrent aspiration [9]. To decrease the risk of aspiration, it is common for patients with FD to undergo early surgical fundoplication and gastrostomy [9]. Consequently, most patients receive nutrition through a combination of solids by mouth and fluids through the gastrostomy tube. The gastrostomy tube also allows feeding and hydration during the hyperadrenergic autonomic crises, during which patients are nauseous, retch profusely and are incapable of oral intake.

Compared to the general population, patients with FD tend to have a lower weight and shorter height, with 75% of FD patients having a low body mass index (BMI) [10]. The cause of this stunted growth is not known. It is possible that frequent vomiting and hospitalizations, renal insufficiency, malabsorption, poor appetite leading to malnutrition, abnormal metabolism and chronic infections may all be contributing factors. FD-causing homozygous *ELP1* mutations may also have a primary role, as genetic mouse models of FD also show marked growth retardation [11].

Monitoring growth is essential to detect growth-hormone deficiency and other potentially treatable symptoms, and to evaluate the response to treatments aimed at increasing BMI. Although disease-specific growth charts are available for some neurological disorders, they have not been developed for the FD population.

Here, we report on the creation of FD-specific growth charts using information from 680 patients collected over four decades. We used these charts to test the hypothesis that lower BMI could be associated with shorter survival of FD patients.

## Materials and methods

We conducted a retrospective review of 680 patients with FD, stored at the NYU FD Registry over a ~50-year period (1970–2020). The NYU FD Registry is an ongoing, prospective registry of patients with FD. The Registry started in 1970 and contains clinical and diagnostic data, including the cause of death, on 680 patients at the time of the present study. All patients have genetically confirmed FD; more than 99% are homozygous for the same mutation (6T>C change) in the *IKBKAP* gene. The majority of patients included in the Registry are from the United States. The remaining patients are from Israel, Canada, United Kingdom, South America, South Africa, Australia, Belgium, Mexico and Germany. Patients are followed closely and seen at least once a year as part of routine clinical follow-up or research-study visits.

### Ethics statement

This study was performed under the guidelines and approval of the NYU Grossman.

School of Medicine IRB under protocol numbers i9153 and i16-01774. All patients signed a written informed consent form agreeing to participate in the registry.

### Growth plots

The data used for the creation of the growth charts were: age at examination (years and months), height (cm), weight (kg), and BMI (kg/m^2^). Growth charts were developed to show the distribution of a measurement (in this case, height, weight and BMI) as it changes with age. Growth charts were smoothed for weight, height, and BMI percentiles, by sex and age. Smoothed line fitting was achieved using Locally Weighted Scatterplot Smoothing (LOWESS) regression [12, 13].

As a comparison group, we used standard global growth curves developed by the Centers for Disease Control and Prevention (CDC) and the World Health Organization (WHO), currently used as global standards to evaluate growth in healthy children [14].

### Statistical analysis

Statistical analysis was performed using R statistical software, RStudio version 1.1.456. One sample t-test was performed to evaluate FD measurements at birth compared to the controls’ mean values. To test the hypothesis that lower BMI was associated with poorer survival, Kaplan-Mayer survival curves (GraphPadPrism9) were obtained by comparing patients with normal BMI (between 19–25 kg/m^2^) to those with BMI below the normal range (<19 kg/m^2^) at the age of 20 (or the closest value after 20 years of age if data at 20 years of age was not available). Furthermore, as weight, but not recumbent length, was significantly different at birth in patients with FD vs the general population, we also obtained survival plots based on weight at birth. Specifically, we compared: i) the survival of patients with weight above the mean vs those with a weight below the mean; and, ii) the survival of patients whose weight was >1 SD from the mean vs patients whose weight was <1 SD from the mean. The latter comparison was intended to highlight any potential differences between groups of individuals at the extremes of weight distribution.

## Results

### FD demographics

Of the 680 patients included in the FD database, height, weight, and BMI were available for 591 patients (Table 1). Females had a slightly higher representation than males (51% vs. 49%).

**Table 1:** Growth demographics in patients with FD. Data presented as mean ± SD.

	Age (years)	Weight (kg)	Height (cm)	BMI (kg/m²)
Males (n = 290, 49%)	18.5±8.7	38.0±16.7	141±28	17.95±3.63
Females (n = 301, 51%)	19.1±9.1	34.8±13.9	136±23	17.84±3.45
All (n = 591)	18.8±8.9	36.4±15.4	138±26	17.89±3.54

### Overall characteristics of growth in patients with FD

**Fig 1:**
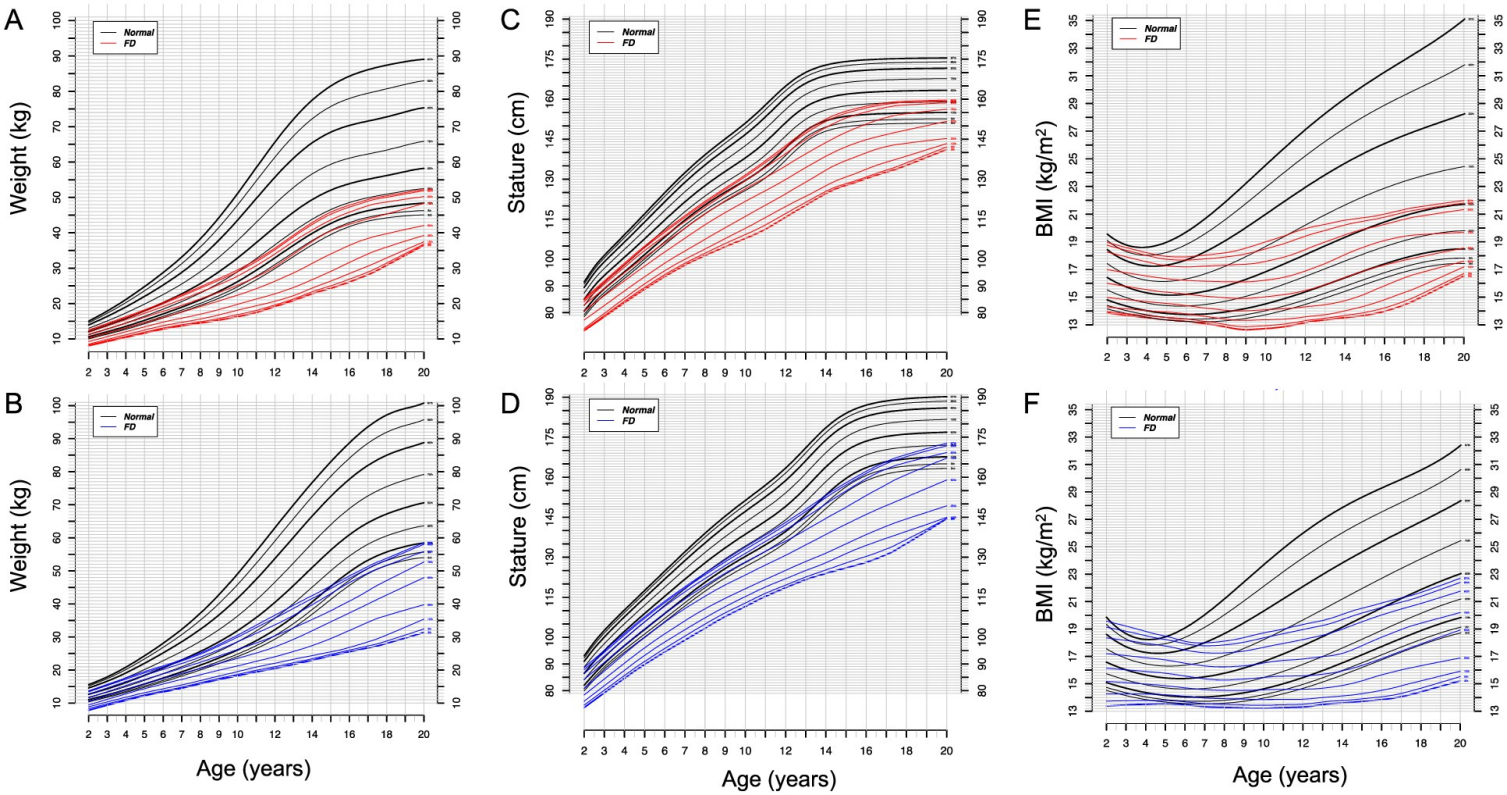
Weight, stature, and body mass index (BMI) for age in patients with familial dysautonomia (FD) aged 2–20 years old. Selected smoothed percentiles. Red plots (A, C, E) denote females (n = 301), blue plots (B, D, F) denote males (n = 290), and black plots (all panels) represent CDC reference charts for the general population.

Compared to normal subjects, patients with FD had markedly delayed growth in both height and weight from an early age. Both males and females with FD grew more slowly and reached a lower adult weight than the general population (Fig 1A and 1B). By the age of 20, the FD 50^th^ percentile corresponds to less than the 3rd percentile of the general population for weight. Similar results were obtained for height (Fig 1C and 1D). In females with FD, the 50^th^ percentile for height corresponds to the 5^th^ percentile of the general population. In males with FD, the 50^th^ percentile of height is below the 3^rd^ percentile of normal. By age 20, all height and weight measurements in FD patients were below the 50^th^ percentile of the general population. This reduced height and weight translates into a reduced BMI for both male and female patients (Fig 1E and 1F). As a result, 76.2% of females and 91.1% of males with FD had BMI measurements below the 50^th^ percentile of the general population.

### Growth after the age of 20

**Fig 2:**
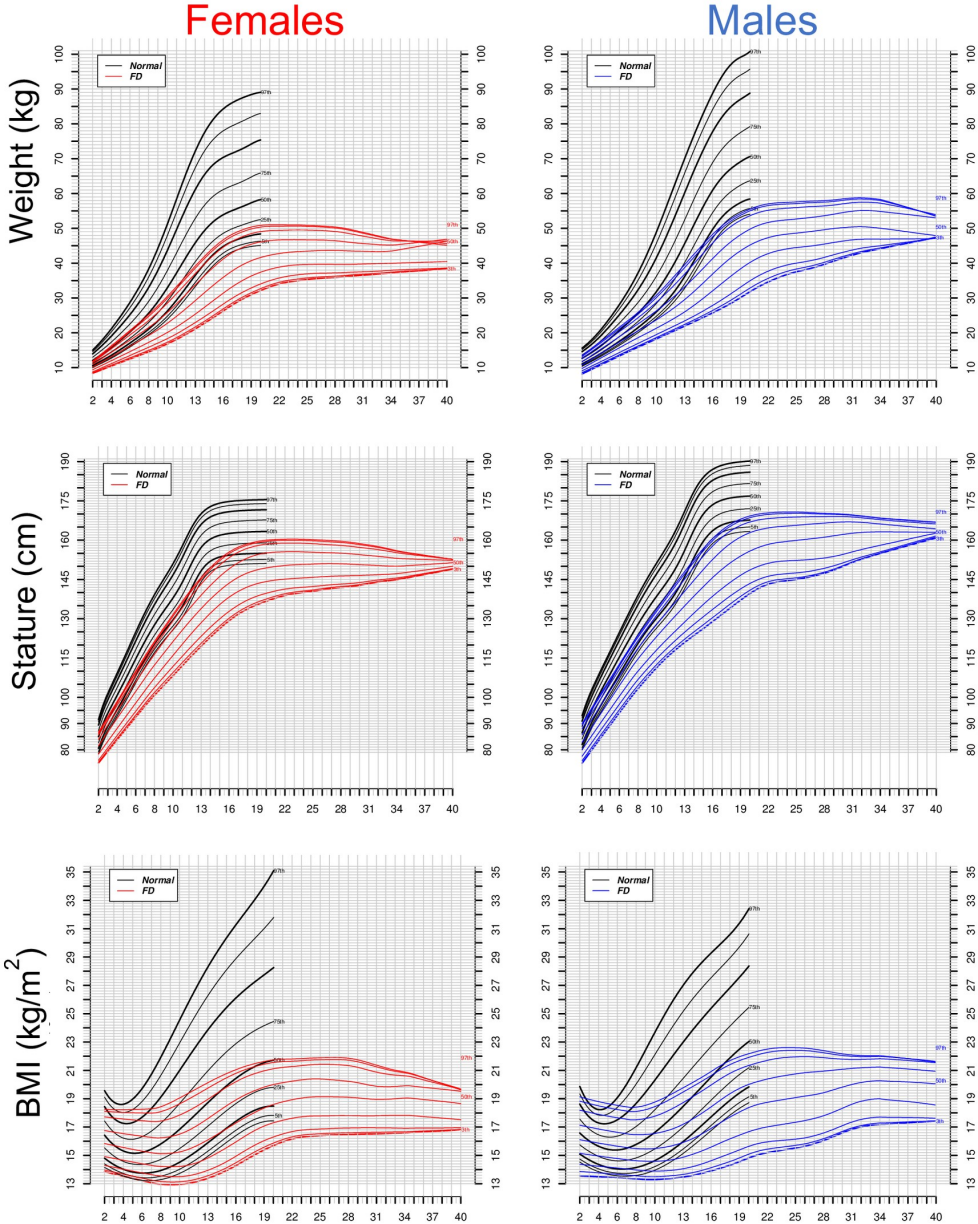
Growth plots for individuals with familial dysautonomia (FD) aged 2–40 years old. Selected smoothed weight (top graphs), height (middle graphs) and body mass index (BMI) (bottom graphs) percentiles for female and male individuals with FD (color lines) compared to control population (black lines). The number of points per interval are as follows. MALES: 226 (0–10); 206 (10–20); 142 (20–30); 64 (30–40). FEMALES: 227 (0–10); 210 (10–20); 148 (20–30); 68 (30–40).

Interestingly, patients with FD experience delayed growth but continue growing in height after the age of 20, unlike the general population. To better understand the FD growth patterns, we developed growth charts from 2 to 40 years of age (Fig 2). These growth charts showed that many individuals with FD continue to grow in stature after the age of 20. Between 22–25 years of age, height reaches a plateau in almost all the percentiles except for the slowest one, which seems to keep growing. The BMI and weight remain stable in FD from the age of 20, in contrast to the general population, which tends to increase their weight with age (Fig 2, top and bottom panels).

To compare the growth of patients with FD vs the normal growth chart, we used the data at age 25 years old to ensure that growth has already reached a plateau. If data at age 25 were not available for a particular patient with FD, the closest age (e.g., 24 years old) was used, but in no case before age 20. At age 25, FD males are taller and heavier than FD females, like the general population (Table 2). The difference in stature between males and females is more pronounced in the FD than in the general population (males are 8.27% taller than females in normal controls vs. 16.17% taller in FD). Conversely, whereas normal males are 21.26% heavier than females, FD males are only 10% heavier than females.

**Table 2:** Growth demographics in patients with FD at or around age 25 and healthy subjects in the 50^th^ percentile at age 20 (CDC). Data presented as mean ± SD.

	Age (years)	Weight (kg)	Height (cm)	BMI (kg/m²)
**Familial dysautonomia**				
Males (n = 147, 48.5%)	25.4±4.0	48.0±10.5	158±11	19.22±3.60
Females (n = 156, 51.5%)	26.3±4.9	43.5±9.2	136±23	19.38±3.25
All (n = 303)	25.9±4.5	45.7±10.1	154±11	19.31±3.42
**Healthy subjects (CDC)**				
Males	20	70.59	176.84	23.04
Females	20	58.21	163.33	21.72

After the age of 20, there was no BMI difference between males and females with FD. This is different from the general population where males have a BMI 6% higher than females at age 20, when height has reached a plateau.

### Weight and length at birth

**Fig 3:**
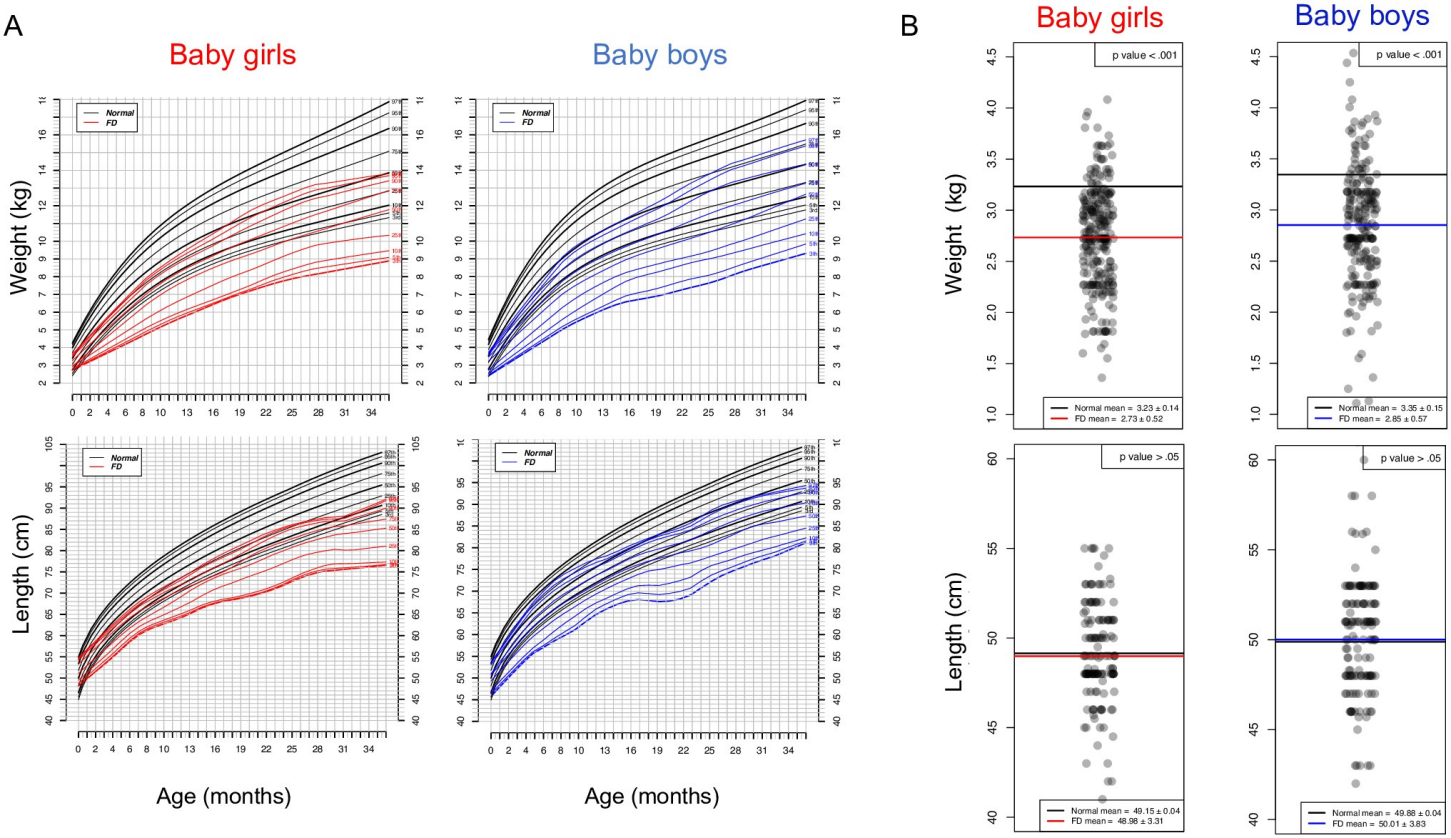
Growth, length, and weight of babies with familial dysautonomia (FD) at birth compared to the general population. A. Growth plots in patients with FD 0–36 months old. Selected smoothed weight (top) and height (bottom) percentiles for female and male babies with FD. B. Length (cm) and weight (kg) of patients with FD at birth compared to the mean at birth of the general population (CDC). Each dot represents one FD patient. The red line indicates the mean weight (top) and length (bottom) at birth of FD baby girls; blue line indicates the mean weight (top) and length (bottom) at birth of FD baby boys. Mean values for healthy baby girls and boys of the general population are indicated by a black line in all plots. P-values are indicated in the right top corner of every graph. N = 143 (FD boys); N = 144 (FD girls) for length; N = 221 (FD boys); N = 258 (FD girls) for weight. For the birth values of the general population, number of subjects is N = 893 for boys and N = 843 for girls for both length and weight [14].

To determine whether growth is already compromised at birth in FD patients, or if the comorbidities associated with FD after birth might be responsible for the delayed growth, we developed growth charts from 0 to 24 months of age (Fig 3A). We also compared the weight and length values at birth between babies with FD and the general population (Fig 3B). There was no difference in the mean recumbent length at birth in FD vs. normal newborns (mean 48.98±3.31 vs 49.15±0.04, p>0.05 for baby girls; 50.01±3.83 vs 49.88±0.04, p>0.05 for baby boys), but the mean weight was significantly lower in FD babies compared to normal babies (2.73±0.52 vs. 3.23±0.14, p < 0.01 in girls and 2.85±0.57 vs 3.35±0.15, p <0.01 in boys).

### Treatments that affect BMI: Growth hormone and spinal fusion surgery

The two most common treatments that FD patients undergo to alter their height and BMI are: growth hormone (GH) administration and spinal fusion surgery.

**Fig 4:**
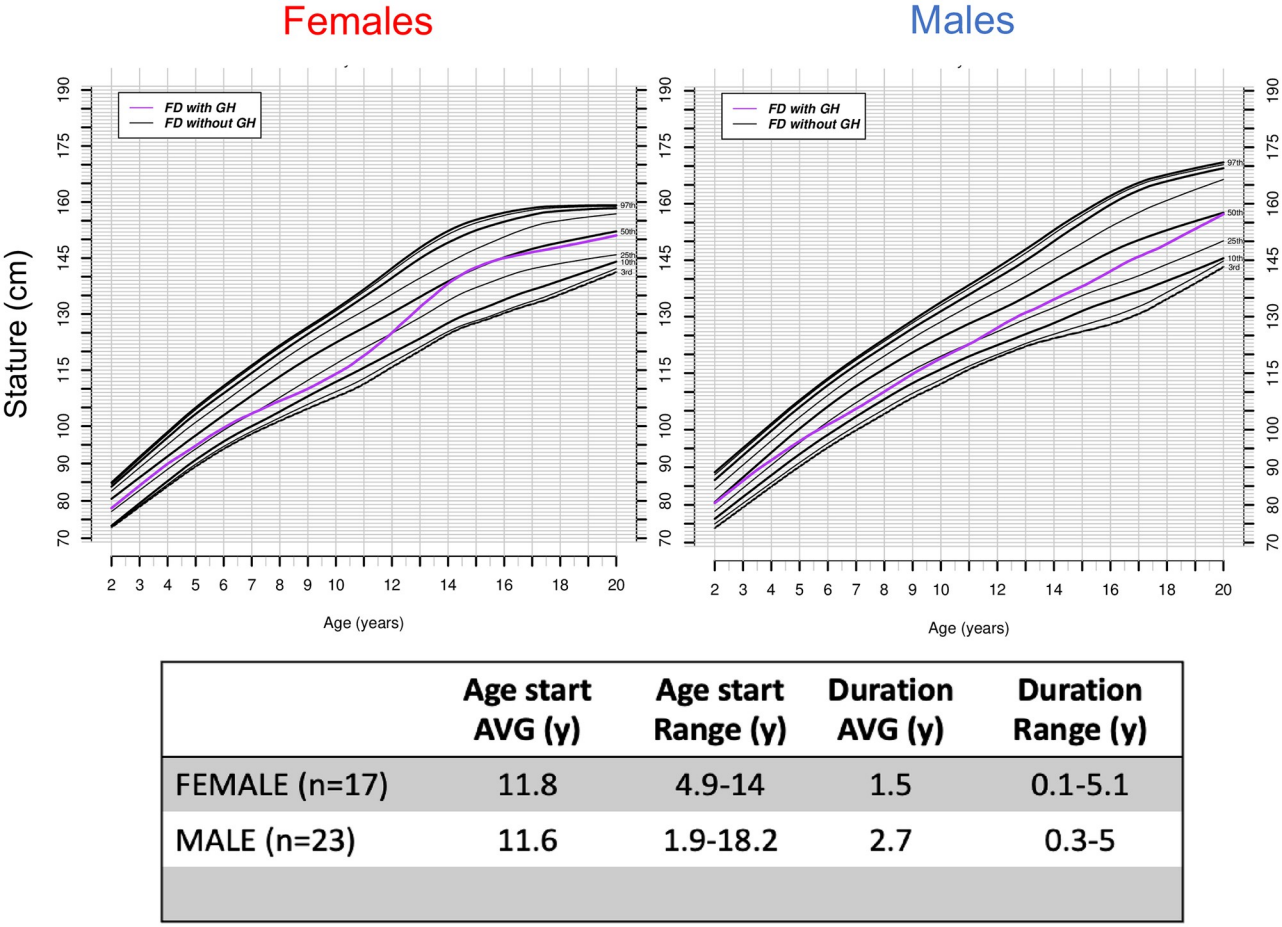
Growth curves for familial dysautonomia FD patients who received growth hormone treatment. Selected smoothed height graphs percentiles for female and male individuals with FD that received GH treatment (color lines; N = 20 males; N = 14 females) compared to FD patients with no GH treatment (black lines; N = 140 males; N = 132 females).

In our database, we found 21 male and 17 (15 with complete data) female patients who had received GH therapy. An earlier analysis of 13 of these patients was published in 2004 [9]. The characteristics and growth pattern of these patients is shown in Fig 4. In both males and females, the age at which GH treatment starts is similar (11.8 years-old in females vs 11.6 years-old in males). When comparing these patients to FD patients without hormone treatment, we can see a jump from the 25^th^ to the 50^th^ growth percentile in both male and females although the effect seems to be more robust in females.

Data in S1 and S2 Tables show the effect of GH treatment in more detail. All female patients in S1 Table show a growth velocity of 5 cm/year or less in the year before starting GH treatment. In most cases, GH treatment improved the growth velocity at least one year after GH started and only in some rare cases the effect extended beyond 12 months after treatment started. Some patients had to discontinue treatment due to side effects.

A similar effect was observed in male FD patients treated with GH (S2 Table). We saw a robust effect in patients who started treatment around age of 10 or less but a much more modest effect if patients started treatment at older ages.

Regarding spinal fusion surgery, as many as 45% of the FD patients underwent this procedure to correct for increasing scoliosis. Table 3 shows the summary of the resulting growth parameters after the procedure. For patients who also underwent spinal fusion surgery, we did not see much difference in the height attained at around 20 years of age or 10 years later.

**Table 3:** Growth, height and weight in patients with FD that underwent spinal fusion surgery. Data presented as mean ± SD.

FD with Spine Fusion	Age (y)	Weight (kg)	Height (cm)	BMI (kg/m²)
Males (n = 50)	~12	26.8±4.7	130±7	15.9±2.1
Female (n = 44)	~12	26.5±6.8	129±10	15.7±2.7
Males (n = 23)	~20	43.7±9.2	155±9	18±3.0
Females (n = 22)	~20	43.0±6.7	150±7	19±2.2
**FD w/o Spine Fusion**				
Males (n = 58)	~12	28.3±1.3	132±10	16.2±2.1
Female (n = 61)	~12	26.6±1.3	128±10	16.2±2.9
Males (n = 29)	~20	45.3±9.9	156±14	18.5±2.6
Females (n = 19)	~20	43.7±6.2	148±9	20.1±3.3

### BMI does not impact survival in patients with FD

**Fig 5:**
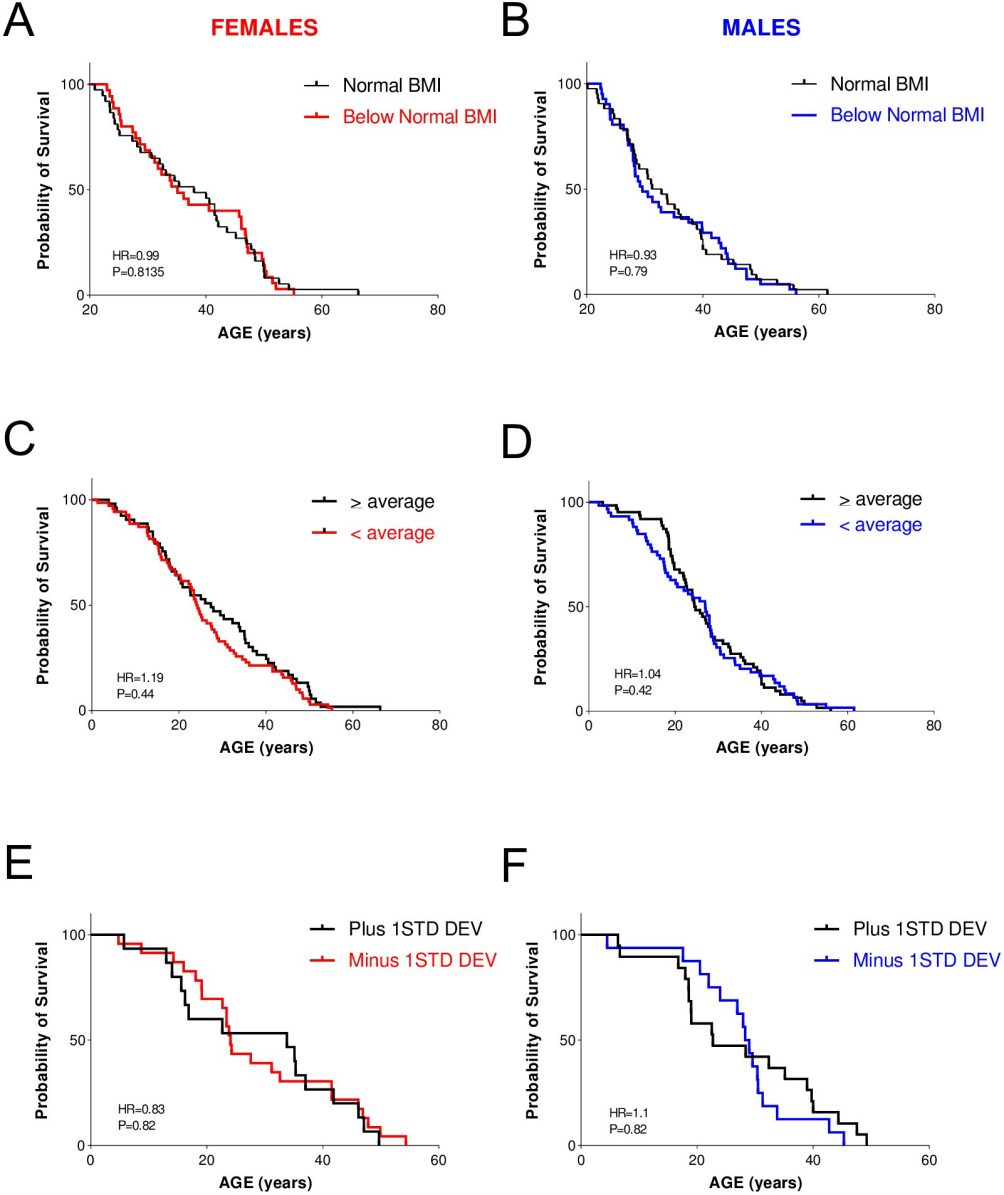
Impact of body mass index and weight at birth on survival in patients with familial dysautonomia (FD). Cumulative probability of survival in patients with FD with normal BMI vs patients with FD with low BMI at age 20 in females (A) and males (B). Cumulative probability of survival in patients with FD with weight at birth at or above average vs patients with FD with weight below average in females (C) and males (D). Cumulative probability of survival in patients with FD with weight at birth at or above 1 SD of the average vs patients with FD with weight below 1 SD of the average in females (E) and males (F).

We investigated whether a higher, closer-to-normal BMI improves the survival of patients with FD. Kaplan-Meier curves disclosed that patients with FD with normal BMI (>19 kg/m^2^) had a similar cumulative probability of survival than patients with FD and low BMI (HR 0.99 P = 0.81 females; HR 0.93 P = 0.79 males) (Fig 5). As weight, but not height, was significantly different in patients with FD compared to the general population, we also performed Kaplan-Meier curves to compare the probability of survival of patients with FD and average or above-average weight at birth vs. patients with FD with below-average weight. This comparison also failed to disclose any significant difference (HR 1.19 P = 0.44 females; HR 1.04 P = 0.42 males). Last, to explore if the survival of FD patients was different as a function of extreme differences in weight at birth, we performed Kaplan-Meier curves comparing patients with FD whose birth weight was greater than 1 SD above the mean vs. patients with FD whose weight was lower than 1 SD below the mean. Again, we found no significant differences in the cumulative probability of survival (HR 0.83 P = 0.82 females; HR 1.1 P = 0.82 males). These results suggest that BMI has no impact on the survival of the FD population.

## Discussion

The growth patterns in patients with FD had not been systematically studied. Before this report, growth charts specific to the FD patient population were not available.

The development of disease-specific growth curves is an important tool for assessing health status and growth in the first 20 years of life and is critical to inform medical decisions and clinical care. In the FD population, they can be particularly valuable because of the multiorgan effects of this disease.

Reduced growth has been described in this population [10], and the question remained whether this difference resulted from the medical fragility that this population endures throughout life, or if there was an inherent failure to thrive directly caused by the *ELP1* mutation.

We found that patients with FD have low birth weight, corresponding to around 85% of birth weight in the general population, but their height is normal. Maximal fetal growth occurs during the last 2 months in utero, especially in weight. It is also during this period that the nervous system has its most significant development in the human embryo. In animal models of FD, this is when progenitor and peripheral neurons regulated by ELP1 start dying.

FD mice always have low birth weights [15, 16]. Although the complete deletion of the mouse *Ikbkap* causes embryonic lethality, a conditional allele generated by Dietrich [17] allowed to analyze FD pups a bit longer. These animals had poor locomotor coordination, unsteady gait and postural inability and hypersensitivity and gastrointestinal dysfunction, all phenotypes consistent with the signs of the disease in FD patients. Importantly, these animals were born with 75–85% the weight and size of their littermates. In addition, the animals that survived were about 32% the weight of their WT littermates at postnatal day 18–21. All these observations argue in favor of an inherent deficit of the mutation that affects growth and weight in the FD patients, and not environmental conditions or medical interventions.

Our statistical analysis did not show reduced height in FD at birth compared to control newborns, but the standard deviation in FD was substantially larger than in controls. This fact reflects the large range of disease severity observed in FD patients.

The inability to reach adequate weight continues later in life in these patients. When height has already reached a plateau after the age of 20, FD individuals still experience difficulties gaining weight. Whereas the general population tends to gain weight (and consequently BMI) at later stages in life, weight and BMI seem to stall in FD patients as they get older.

Our growth curves show that people with FD experience delayed growth from very early in life, even before birth. Growth is slower than in the average population, and the final weight and height attained are lower. By the age of 20, most people with FD fall below the 50th percentile of the general population in terms of height and weight, and half fall below the bottom 5th percentile. However, the height of a person with FD reaches a plateau around age 25, compared to age 20 in the general population. The exact age at which height in FD reaches a plateau was difficult to assess given our small sample size; although we have data points at different ages around that age, we do not have these at the exact same age for all patients. Also, weight appears to remain more stable after age 20 than in the general population, in which weight tends to increase with age. This contributes to a lower BMI in patients with FD in adulthood.

Another observation from our growth analysis is that there may be some sex-associated differences in the regulation of growth. The differences in height and weight between males and females do not follow the same pattern as in the general population. Males with FD are 16% taller than females with FD in adulthood, twice the difference in height between males and females in the general population (8.27%). This means that females have more difficulty reaching normal height than males with FD, but both males and females have difficulties to maintaining weight at all stages in life.

Our analysis of the effect of treatments that improve height showed that GH had variable effects depending on age at the start and duration of treatment, although our data confirm earlier observations by Kamboj et al. [10] that growth hormone may help FD patients in attaining their expected growth. It is important to note, though, that GH does not appear to contribute to a significant jump in growth percentile outside the FD growth plots. A similar conclusion can be drawn from the data summarized for spinal fusion intervention.

We also attempted to establish whether an improved BMI, a measure of height and weight, can improve survival in patients with FD. BMI is routinely used in the clinic to evaluate the overall health of patients. While having a high BMI due to obesity has known implications for health and survival, the mortality risks of being underweight and, by extension, of lower BMI cannot be ignored. Several reports have found a J-shaped association between BMI and overall mortality with increased hazard ratios with BMIs lower than 18.5 [17, 18] and stronger associations seen at younger than at older ages [18]. In the context of other diseases, malnutrition, and low BMI are correlated with a higher risk of dementia and mortality [19]. Lower BMI is also associated with worse pulmonary function and reduced survival in Cystic Fibrosis (CF) [20, 21], a population with important similarities to our FD patients in terms of respiratory function.

In our analysis, we found no significant difference in the survival curves between individuals with normal or close to normal BMI and those with lower BMIs. Similarly, we found no impact on survival if individuals were born with a lower weight. This suggests that a smaller BMI in patients with FD is not contributing to early death.

We hope that the development of FD-specific growth curves will help in the evaluation of nutritional or other interventions that might improve growth and overall health. This is especially important in a population like FD, given the reduced growth that characterizes this patient population. It is critical to establish a close collaboration between the neurological and nutritional teams of FD patients to ultimately determine the best growth outcomes for FD patients.

## Supporting information

S1 Data (XLSX)

S1 Table Growth Hormone treatment in female patients with FD. Growth velocity (GV in cm/year) of female FD patients one year before and 1–5 years after treatment start. Height 1 indicates the height around 20 years of age or the closest value. Height 2 indicates the height at 30 years of age or closest value. n/a, not applicable (patient too young and/or still alive); unk, unknown (missing data); *, spine fusion surgery.
(DOCX)

S2 Table Growth Hormone treatment in male patients with FD. Growth velocity (GV in cm/year) of male FD patients one year before and 1–5 years after treatment start. Height 1 indicates the height around 20 years of age or the closest value. Height 2 indicates the height at 30 years of age or closest value. n/a, not applicable (patient too young and/or still alive); unk, unknown (missing data); *, spine fusion surgery.
(DOCX)
