# Supplementary material for: Height, weight, and body mass index in patients with familial dysautonomia
Source: PLoS One. 2023 Nov 9;18(11):e0293800. doi: 10.1371/journal.pone.0293800 (PMC10635437; doi:10.1371/journal.pone.0293800)
Supplement: S1 Table — Growth velocity (GV in cm/year) of female FD patients one year before and 1–5 years after treatment start. Height 1 indicates the height around 20 years of age or the closest value. Height 2 indicates the height at 30 years of age or closest value. n/a, not applicable (patient too young and/or still alive); unk, unknown (missing data); *, spine fusion surgery. (DOCX) [file pone.0293800.s002.docx]

**S1 Table.**

| Patient | GH starts (age) | GH duration (years) | GV year before (cm/year) | GV 1y after  (cm/year) | GV 2y after  (cm/year) | GV 3y after  (cm/year) | GV 4y after  (cm/year) | GV 5y after  (cm/year) | Side Effects | Height 1 (AGE) | Height 2 (AGE) | AGE at  death |
| --- | --- | --- | --- | --- | --- | --- | --- | --- | --- | --- | --- | --- |
| F1 | 10 | 1 | unk | unk | unk | unk | unk | unk | Tumor in thigh | 1.549 (28) | 1.581  (38) | 41.6 |
| F2 | 10.3, 14 | ? | 5 | 3.73 | 2.26 | 2.5 | -0.26 | 0.05 |  |  |  | 36 |
| F3 | 12 | 2.7 | unk | unk | unk | unk | unk | unk |  | 1.422 (20) |  | 22 |
| F4* | 13.8 | 3.8 | 2.6 | 8.7 | 4 | 4.73 | 2.6 | 0.42 | Headaches | 1.65 (20) | 1.61 (30.6) | n/a |
| F5* | 13.8 | 2.9 | 5.1 | 5 | 3.45 | 2.5 | 0.22 | 1.13 |  | 1.49 (20.5) | 1.5 (29.3) | n/a |
| F6 | 12.3 | 0.1 | 4.4 | 4 | 3.75 | 4 | 0 | 3.8 | Excessive weight gain | 1.448 (20.5) | 1.44 (27.7) | n/a |
| F7* | 11.3 | 5.2 | 3.19 | 6.3 | 7 | 5.70 | 7.5 | 4.0 |  | 1.581 (19.6) | 1.56 (27.5) | n/a |
| F8* | 11.3, 13.5 | 0.4  0.2 | 3.2 | 3.17 | 4.22 | 5.1 | n/a | n/a | Headache,  increased crises | 1.321 (15) | n/a | 15 |
| F9 | 9.1 | 5.1 | unk | unk | 5.06 | unk | unk | 4.73 |  | 1.435 (19) | 1.44 (24.9) | n/a |
| F10 | 12.2 | 1.2 | 3.8 | 7.35 | 7.35 | 1.7 | 3.5 | 0.36 |  | 1.5 (20.5) | n/a | n/a |
| F11* | 12.5 | 0.5 | 3.67 | 5.1 | 4.10 | 2.73 | 0.67 | 0.10 |  | 1.378 (19.4) | 1.39 (29.4) | n/a |
| F12 | 4.9 | ? | 4.3 | 8.2 | 5.3 | 6.2 | 6.5 | 7.7 |  | 1.465 (19.4) | n/a | n/a |
| F13 | 10.8 | ? | 2.69 | 12.22 | 8.46 | 7.11 | unk | 2.93 |  | 1.524 (19.6) | n/a | n/a |
| F14 | 12.2 | ? | 5.45 | unk | 3.33 | unk | 4.94 | unk |  | 1.51 (18.5) | n/a | n/a |
| F15 | 7.2 | ongoing | 2.25 | 5.09 | 5.5 | 5.38 | 4.6 | 3.27 |  | 1.16 (12.5) | n/a | n/a |
