# Supplementary material for: Height, weight, and body mass index in patients with familial dysautonomia
Source: PLoS One. 2023 Nov 9;18(11):e0293800. doi: 10.1371/journal.pone.0293800 (PMC10635437; doi:10.1371/journal.pone.0293800)
Supplement: S2 Table — Growth velocity (GV in cm/year) of male FD patients one year before and 1–5 years after treatment start. Height 1 indicates the height around 20 years of age or the closest value. Height 2 indicates the height at 30 years of age or closest value. n/a, not applicable (patient too young and/or still alive); unk, unknown (missing data); *, spine fusion surgery. (DOCX) [file pone.0293800.s003.docx]

**S2 Table**

| Patient | GH starts (age) | GH duration (years) | GV year before (cm/year) | GV 1y after  (cm/year) | GV 2y after  (cm/year) | GV 3y after  (cm/year) | GV 4y after  (cm/year) | GV 5y after  (cm/year) | Side Effects | Height 1 (AGE) | Height 2 (AGE) | AGE at  death |
| --- | --- | --- | --- | --- | --- | --- | --- | --- | --- | --- | --- | --- |
| M1 | 15.7 | 0.3 | 4.4 | 16.18 | n/a | n/a | n/a | n/a | Edema, hypertension, seizure, vomiting | 1.448 (16.3) | n/a | 17.3 |
| M2* | 17.4 | 0.3 | 2.5 | 3.12 | 3.45 | 3.55 | 0 | 0.46 | edema | 1.588 (19.9) | 1.577 (28.1) | n/a |
| M3* | 15.8 | 4.1 | 6.33 | 1.79 | 1.79 | 1.79 | unk | unk |  | 1.638 (19.9) | 1.63 (38.3) | n/a |
| M4* | 15.3 | 4.3 | 0 | 4.64 | 4.5 | 4.27 | unk | unk |  | 1.664 (22.3) | n/a | n/a |
| M5* | 12 | unk | 3.2 | unk | unk | unk | unk | 5.09 | Shaking, increased crises | 1.435 (17.2) | n/a | 23 |
| M6 | 14.6 | 0.3 | unk | 5.84 | 5.84 | 9 | unk | unk |  | 1.62 (20.7) | 1.63 (37) | 38 |
| M7* | 6.6 | 2.2 | unk | unk | 7.08 | unk | 3.8 | 3.5 |  | 1.56 (27.3) | 1.575 (40) | n/a |
| M8* | 9.7 | 1 | 4.5 | 9.22 | 2.4 | 3.92 | 2.60 | 2.40 |  | 1.543 (20.3) | 1.6 (27.6) | 28 |
| M9* | 10.3 | 5 | 5.93 | 7.55 | unk | 5.47 | 5.67 | 7 |  | 1.695 (22.1) | 1.705 (31.4) | n/a |
| M10* | 12.1 | 4.6 | 2.89 | 5.1 | 6.90 | 6.00 | 9.38 | 4.37 |  | 1.702 (19.5) | 1.725 (26.2) | n/a |
| M11* | 14 | 2.3 | unk | 5.7 | 4 | -0.75 | 4.64 | -1.18 |  | 1.461 (20.1) | 1.48 (34.9) | n/a |
| M12 | 18.2 | unk | 0.7 | -0.9 | 0 | unk | unk | unk |  | 1.42 (20.3) | n/a | 21 |
| M13 | 12.3 | unk | unk | unk | 5.63 | unk | unk | unk |  | 1.5 (14.2) | n/a | n/a |
| M14 | 12.9 | unk | 0.32 | 0.21 | 0.32 | 0.42 | 0.35 | 0.34 |  | 1.555 (21) | 1.54 (29.3) | n/a |
| M15* | 14.2 | 4.1 | 4 | 4.6 | 8.33 | 3.20 | 3.10 | unk |  | 1.65 (19.9) | 1.62 (32.5) | n/a |
| M16 | 6.6 | 2.3 | 4.44 | 8 | 5.71 | 8 | 3.12 | 2.65 |  | 1.44 (20.4) | n/a | n/a |
| M17 | 9.3 | unk | 3.2 | 4.5 | 4.4 | 2.77 | 2.11 | 5.4 |  | 1.448 (20.1) | n/a | n/a |
| M18 | 11.4 | 0.4 | 3.09 | 4.84 | 3.92 | 1.82 | 4.22 | 3.00 |  | 1.5 (21) | n/a | n/a |
| M19 | 10.7 | 3.2 | 5.58 | 6 | 4.9 | 2.17 | 1.73 | 5.04 |  | 1.473 (19.4) | n/a | n/a |
| M20 | 11.5 | unk | unk | unk | 6.29 | unk | 4.82 | 5.20 |  | 1.6 (20) | n/a | n/a |
| M21 | 10.7 | ongoing | 4.92 | 5.33 | 8.18 | n/a | n/a | n/a |  | n/a | n/a | n/a |
